# Diagnostic Accuracy of the Overlapping Infinity Loops, Wire Cube, and Clock Drawing Tests for Cognitive Impairment in Mild Cognitive Impairment and Dementia

**DOI:** 10.1155/2017/5289239

**Published:** 2017-01-31

**Authors:** Thammanard Charernboon

**Affiliations:** Division of Clinical Epidemiology and Department of Psychiatry, Faculty of Medicine, Thammasat University, Pathum Thani, Thailand

## Abstract

*Purpose*. To investigate the diagnostic accuracy of the overlapping infinity loops, wire cube, and clock drawing tests (CDT) in the detection of mild cognitive impairment (MCI) and dementia.* Method*. The participants were 60 normal controls (NC), 35 patients with MCI, and 47 patients with mild dementia.* Results*. The results illustrate that infinity loops, cube, or CDT were not able to discriminate between NC and MCI groups. In dementia detection, the CDT had the highest diagnostic accuracy (sensitivity 76.6% and specificity 87.4%) followed by infinity loops (sensitivity 63.8% and specificity 91.6%) and cube (sensitivity 93.6% and specificity 46.3%).* Conclusion*. This study demonstrates that the three drawing tests are sensitive detectors of dementia but not MCI.

## 1. Introduction

The wire cube and clock drawing tests (CDT) have been the most frequently used figure drawing tests in cognitive function assessments. Both items embrace visuospatial constructional abilities and executive function. Many cognitive assessment tools make use of the wire cube and CDT as parts of cognitive screening tests, for example, the Montreal Cognitive Assessment (MoCA), the Rowland Universal Dementia Assessment Scale (RUDAS), and the Addenbrooke's Cognitive Examination (ACE) [1–3]. Additionally, the CDT alone is also widely used in screening for cognitive impairment and dementia since studies showed that it has good diagnostic accuracy for dementia; some studies also suggest that the normal CDT can reasonably exclude cognitive impairment in dementia [4].

On the other hand, the overlapping infinity loops is a rather new figure copying test. It has been used in the Addenbrooke's Cognitive Examination version III in 2013 [1]. It is expected to replace the use of overlapping pentagons due to the Mini-Mental State Examination (MMSE) copyright enforcement which includes the intersecting pentagons [5]. Therefore, the overlapping infinity loops' diagnostic properties are scant.

Although these drawing tests have been frequently used, most studies examined their properties in people with dementia [4, 6]. Studies in mild cognitive impairment (MCI) are limited, and only a few studies have compared the diagnostic accuracy of these tests together.

The objective of this study was to determine the diagnostic accuracy of the three figure drawing tasks in the detection of MCI and dementia.

## 2. Materials and Methods

The study was conducted at Thammasat University Hospital, Thailand, from January 2014 to January 2016. The participants were at least 55 years old; those with visual/hearing impairment, serious neurological, or psychiatric disorders (e.g., schizophrenia) were excluded. There were 60 normal controls (NC), 35 patients with MCI, and 47 patients with dementia. People with dementia and MCI were recruited from the Memory Clinic, and NC were recruited from the patients' relatives. The study was approved by the Human Ethics Committee of Thammasat University (protocol number: MTU-EC-PS-6-019/57).

### 2.1. Diagnosis

Diagnosis of dementia was based on the Diagnostic and Statistical Manual of Mental Disorders (DSM-5) criteria for a major neurocognitive disorder [7] by expert psychiatrists at the Memory Clinic. All dementia patients underwent standard diagnostic protocol for dementia, that is, psychological and neurological examination, laboratory examination, and magnetic resonance imaging (MRI). For cognitive assessment, the Thai Mental State Examination (TMSE) [8] was administered. Only mild cases of dementia were chosen to be used in this study. To ensure that people with moderate or severe dementia had not entered the study, participants with a TMSE score below 15 were excluded.

The diagnosis of MCI was established according to the DSM-5 criteria for a mild neurocognitive disorder by expert psychiatrists: (1) modest decline in cognitive function, (2) a modest impairment in cognitive performance (documented by a score of 1.5 standard deviations below the mean normal cognitive elderly score on the Montreal Cognitive Assessment (MoCA) [9]), and (3) cognitive deficits that do not interfere with independent capacity for independence in everyday activities. All patients with MCI received standard diagnostic protocol for MCI of our Memory Clinic, that is, psychological and neurological examination and laboratory examination.

The cognitively healthy elderly participants had exhibited no decline in cognitive function, had normal cognitive functions (according to the MoCA), and were independent in their activities of daily living.

### 2.2. Scoring System

**Figure 1:**
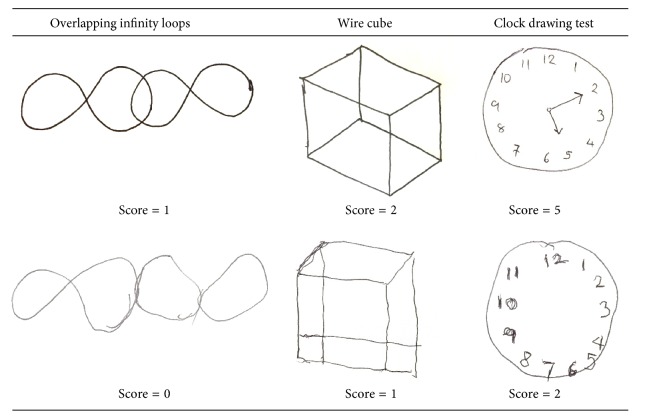
Overlapping infinity loops, wire cube, and clock drawing tests.

The scoring methods for all tests were adopted from the scoring system in the ACE [1]. For the overlapping infinity loops and wire cube tests, the participants were told to copy the figures from the examples. Intersecting infinity loops have a score range of 0 to 1 (0 points if incorrect and 1 if correct). The wire cube has a score range of 0 to 2: 2 points for correct cube, 1 for an incomplete cube (the cube has fewer than 12 lines, but a general cube shape is maintained), and 0 points awarded for incorrect cube (Figure 1).

For the CDT, the participants were asked to draw a clock face with numbers on it with the hands at ten past five. The clock drawing test has a score range of 0 to 5: 1 point for a circle, 1 point for all numbers, 1 point if all numbers are well distributed within the circle, and 2 points if both hands are well drawn and placed on the correct numbers (1 point if only one hand is placed on the correct number).

Demographic and clinical data were collected by the author. The three tests were administered by independent psychologists or psychiatrists.

### 2.3. Statistical Analysis

Demographic data were compared between the participants using exact test and one-way ANOVA with Bonferroni post hoc analysis. We determined how well the three drawing tests distinguished MCI from cognitively normal elderly and further distinguished dementia from nondementia (consisting of normal controls and MCI together) using area under receiver operating characteristic (AuROC), sensitivity, specificity, and likelihood ratio if test positive (LR+). *p* values of less than 0.05 were considered to indicate statistical significance.

## 3. Results

Table 1 shows demographics and scores. The three groups did not differ in terms of gender and educational level. Only age was significantly different between NC versus dementia and MCI versus dementia. The dementia group consists of 30 people with Alzheimer's disease (63.8%), 10 with mixed type (21.3%), and 7 with vascular dementias (14.9%).

NC showed difficulties in drawing a wire cube, evidenced by 46.7% failure, while only 3.3% failed in the overlapping infinity loops. The failure rates increased for those with MCI and increased markedly for dementia groups. Only 36.2% of dementia patients could draw the infinity loops and 6.4% for the wire cube. Regarding the CDT, NC had a mean score of 4.5 (0.9), indicating a complete drawing or only minor error, whereas the dementia group had only 1.9 (1.3) points.

In Tables 2 and 3, we presented the AuROC, sensitivity, specificity, and LR+ of each test in the detection of MCI and dementia. The results illustrate that overlapping infinity loops, wire cube, or CDT alone were not able to discriminate between NC and MCI groups (Table 2).

On the other hand, ROC analyses for detection of dementia showed that all tests have discriminative capacities. The CDT had the highest diagnostic accuracy (AuROC 0.82, sensitivity 76.6%, and specificity 87.4%) followed by overlapping infinity (AuROC 0.78, sensitivity 63.8%, and specificity 91.6%) and wire cube (AuROC 0.7, sensitivity 93.6%, and specificity 46.3%) (Table 3).

## 4. Discussion

The study compared the diagnostic accuracy and the implications of the overlapping infinity loops, wire cube, and CDT in screening MCI and dementia.

There are many scoring systems for the wire cube and CDT ranging from the very simple (right/wrong) to rather detailed (e.g., 18 points scoring for CDT or 20 points for wire cube) [6, 10]. The more complex scoring systems usually require training and take time; a review suggested that increasing the complexity of scoring systems in the CDT only subtly improves the test's accuracy [10]. Therefore, as this study was aimed at the primary care setting or general practitioners, the simple scoring method used in the ACE that gives 1 point for overlapping infinity loops, 2 points for wire cube, and 5 points for the CDT was used.

Notably, the study demonstrated that many cognitively normal elderly participants showed some difficulties in copying a wire cube. Only 53.3% of cognitively normal elderly can copy the wire cube correctly; nevertheless, it is comparable with other studies that only 34–60% of normal elderly could copy the wire cube [6, 11]. Therefore, impairment in wire cube copying does not imply cognitive impairment as indicated by low specificity (46.3–53.3%). In contrast, most normal controls were able to draw overlapping infinity loops and CDT correctly.

Regarding MCI detection, participants with MCI had only slightly lower scores than NC in all three drawing tests leading to low sensitivity ranging from the lowest 17.1% (overlapping infinity loops) to the highest 65.7% (wire cube). These results suggest that the tasks are not sensitive detectors of cognitive impairment in MCI. Consequently, using these tasks alone are not recommended for screening of MCI. This result confirms other studies, such as Powlishta [12], that state that the CDT does not appear to be a valid screening tool for detecting MCI. It can only distinguish between normal aging and at least mild dementia.

In dementia detection, the CDT showed good sensitivity (76.6%) and specificity (87.4%) which were comparable to the previous studies that found that most CDT tasks achieved sensitivity and specificity of approximately 85% [13]. Comparing the three tasks, the CDT had the highest diagnostic accuracy. However, it was quite surprising that overlapping infinity loops seem to be better than the wire cube since it had a significantly higher specificity (91.6% versus 46.3%), but lower sensitivity (63.8% versus 93.6%). This study suggests that overlapping infinity loops could be a good simple figure copying task for detection of cognitive impairment in dementia.

It should also be recognized that although the CDT had the highest diagnostic accuracy for detecting dementia, the sensitivity of 76.6% would yield many false-negative cases which leads to its inability to be used as a screening test when used alone.

In terms of limitations, because most of the dementia participants received a diagnosis of Alzheimer's disease, this diagnostic accuracy may not be applied to other types of dementia, for example, frontotemporal dementia.

## 5. Conclusion

This study demonstrates that overlapping infinity loops, wire cube, and clock drawing test are sensitive detectors of cognitive impairment in dementia but not MCI.

## Figures and Tables

**Table 1:** Characteristics.

	Nondementia		Dementia (*n* = 47) *N* (%)	Differences between groups
	NC (*n* = 60): *N* (%)	MCI (*n* = 35) *N* (%)		
Gender (male)	17 (28.3)	14 (40)	13 (27.7)	ns
Age (years): mean (SD)	66.2 (7.0)	69.9 (8.1)	74.7 (8.9)	b^*∗∗∗*^, c^*∗∗*^
Years of education: mean (SD)	9.7 (5.2)	9.2 (5.4)	8.7 (4.9)	ns
TMSE score	26.9 (2.1)	—	17.2 (3.2)	b^*∗∗∗*^
MoCA score	25.3 (2.2)	18.9 (2.6)	—	a^*∗∗∗*^
Infinity loops score				a^*∗*^, b^*∗∗∗*^, c^*∗∗∗*^
0 (incorrect)	2 (3.3)	6 (17.1)	30 (63.8)	
1 (correct)	58 (96.7)	29 (82.9)	17 (36.2)	
Cube score				a^*∗*^, b^*∗∗∗*^, c^*∗∗*^
0 (incorrect)	0 (0)	8 (22.9)	23 (48.9)	
1 (partial correct)	28 (46.7)	15 (42.9)	21 (44.7)	
2 (correct)	32 (53.3)	12 (34.2)	3 (6.4)	
Clock drawing test: mean (SD)	4.5 (0.9)	3.7 (1.3)	1.9 (1.3)	a^*∗∗*^, b^*∗∗∗*^, c^*∗∗∗*^

ns = nonsignificant; NC = normal controls; MCI = mild cognitive impairment; TMSE = Thai Mental State Examination; MoCA = Montreal Cognitive Assessment.

a = NC versus MCI; b = NC versus dementia; c = MCI versus dementia.

^*∗*^ *p* < 0.05, ^*∗∗*^*p* < 0.01, ^*∗∗∗*^*p* < 0.001.

**Table 2:** Cut-off score, area under receiver operating characteristic (AuROC) curve, and diagnostic accuracy of three drawing tests for detection of mild cognitive impairment.

	Cut-off score	AuROC [95% CI]	Sensitivity [95% CI]	Specificity [95% CI]	LR+ [95% CI]
Infinity loops (0-1)	0	0.57 [0.5–0.64]	17.1% [6.6–33.6]	96.7% [88.5–99.6]	5.1 [1.1–24.1]
Cube (0–2)	≤1	0.65 [0.49–0.7]	65.7% [47.8–80.9]	53.3% [40.0–66.3]	1.4 [1.0–2.0]
CDT (0–5)	≤4	0.64 [0.53–0.74]	57.1% [39.4–73.7]	70.0% [56.8–81.2]	1.9 [1.2– 3.1]

AuROC: area under receiver operating characteristic; LR+: likelihood ratio if test positive; CDT: the clock drawing test.

**Table 3:** Cut-off score, area under receiver operating characteristic (AuROC) curve, and diagnostic accuracy of three drawing tests for detection of dementia from nondementia.

	Cut-off score	AuROC [95% CI]	Sensitivity [95% CI]	Specificity [95% CI]	LR+ [95% CI]
Infinity loops (0-1)	0	0.78 [0.7–0.85]	63.8% [48.5–77.3]	91.6% [84.1–96.3]	7.6 [3.8–15.2]
Cube (0–2)	≤1	0.7 [0.67–0.76]	93.6% [82.5–98.7]	46.3% [36.0–56.8]	1.7 [1.4–2.1]
CDT (0–5)	≤2	0.82 [0.75–0.89]	76.6% [62.0–87.7]	87.4% [79.0–93.3]	6.1 [3.5–10.5]

AuROC: area under receiver operating characteristic; LR+: likelihood ratio if test positive; CDT: the clock drawing test.
